# Structured analysis of the impact of fetal motion on phase-contrast MRI flow measurements with metric optimized gating

**DOI:** 10.1038/s41598-022-09327-1

**Published:** 2022-03-30

**Authors:** Alexander Schulz, David F. A. Lloyd, Milou P. M. van Poppel, Thomas A. Roberts, Johannes K. Steinweg, Kuberan Pushparajah, Joseph V. Hajnal, Reza Razavi

**Affiliations:** 1 School of Biomedical Engineering and Imaging Sciences, King’s College London, St. Thomas’ Hospital, London, UK; 2 Department of Congenital Heart Disease, Evelina London Children’s Hospital, Guy’s and St Thomas’ NHS Foundation Trust, London, UK; 3 Charité – Universitätsmedizin Berlin, corporate member of Freie Universität Berlin and Humboldt-Universität zu Berlin, Charitéplatz 1, Berlin, 10117 Germany

**Keywords:** Biomedical engineering, Congenital heart defects

## Abstract

The impact of fetal motion on phase contrast magnetic resonance imaging (PC-MRI) with metric optimized gating (MOG) remains unknown, despite being a known limitation to prenatal MRI. This study aims to describe the effect of motion on fetal flow-measurements using PC-MRI with MOG and to generate a scoring-system that could be used to predict motion-corrupted datasets at the time of acquisition. Ten adult volunteers underwent PC-MRI with MOG using a motion-device to simulate reproducible in-plane motion encountered in fetuses. PC-MRI data were acquired on ten fetuses. All ungated images were rated on their quality from 0 (no motion) to 2 (severe motion). There was no significant difference in measured flows with in-plane motion during the first and last third of sequence acquisition. Movement in the middle section of acquisition produced a significant difference while all referring ungated images were rated with a score of 2. Intra-Class-Correlation (ICC) for flow-measurements in adult and fetal datasets was lower for datasets with scores of 2. For fetal applications, the use of a simple three-point scoring system reliably identifies motion-corrupted sequences from unprocessed data at the time of acquisition, with a high score corresponding to significant underestimation of flow values and increased interobserver variability.

## Introduction

Fetal cardiac MRI has been shown to be a useful adjunct to ultrasound in the diagnosis of congenital heart disease (CHD) in-utero^1,2^. The additional assessment of fetal blood flow and circulation using phase contrast MRI (PC-MRI) has also been used to explore the relationship between CHD and other organs such as the placenta and the developing fetal brain^3^.

ECG-gated PC-MRI is proven to be an accurate and reliable means of measuring intravascular flows in adults^4–6^. Different approaches have been described to address the lack of ECG gating in fetal life including “metric optimised gating” (MOG)^7^, Doppler ultrasound gating^8^, real-time imaging^9^ and cardiac self-gating^10^. Two of the approaches have been used for fetal cardiovascular flow measurements: the first, MOG, is a retrospective method detecting the heart rate (HR) on oversampled data and is a freely available open-source software^7,11^. This post-processing method leverages temporally oversampled k-space data, by applying hypothetical triggers to the data while iteratively analysing the level of misgating artifact i.e., the cardiac phase error between the true cardiac phase and the phase based on incorrect gating. The reconstruction with the optimal image metric is assumed to reflect the true underlying HR at acquisition^7^. Various validation experiments support the use of MOG as a reference standard for further inventions^12–16^. A second approach, which requires the use of a bespoke Doppler ultrasound gating device, has also been described^8,17^. A comparison of both gating methods for fetal blood flow measurements, found good overall agreement using this method^13^. Arguably however, uncontrolled fetal motion during acquisition remains the most significant limitation of fetal PC-MRI with MOG in practice. In adults, motion during MRI-Scans can clearly be anticipated during acquisition and its effects on conventionally gated PC-MRI and k-space have been described^4,18,19^. Unlike postnatal imaging, motion during fetal PC-MRI sequences can be difficult to identify at the time of acquisition as the fetus is not visible and the data often requires significant post-processing before analysis. This can increase scan and post-processing times. No studies to date have attempted to quantify the effects of fetal motion on the accuracy and reliability of PC-MRI with MOG. This means a potential for high failure rates and/or inaccurate flow measurement following MOG processing.

The purpose of this study is to assess the impact of fetal motion on the accuracy of fetal PC-MRI flow measurements using MOG in an in-vivo model by creating artificial in-plane motion, and to assess a novel scoring system designed to predict the usability and reliability of flow at the point of acquisition.

## Materials and methods

### Study cohort

The study was performed in two parts. First, a controlled “simulated motion experiment” was performed on the neck vessels of healthy adult volunteers, to develop and validate a motion scoring system for PC-MRI data. For this experiment, 10 healthy adult volunteers were recruited under the REC: 01/11/12/ approved by the London Bridge Research Ethics Committee. None of the adult volunteers had any surgery on the neck vessels prior to the scan. For the second part, 10 singleton pregnant women were prospectively recruited for MRI following informed consent as part of a long-running fetal imaging study (REC 14/LO/1806 approved by the same Ethics Committee as above and REC 07/H0707/105 approved by West London & GTAC Research Ethics Committee), in order to assess the impact of this scoring system on overall PC-MRI reliability. The study was conducted in accordance with the Declaration of Helsinki guidelines and all patients in this study provided informed consent.

### Procedure

All data were acquired on a 1.5 T Ingenia MRI scanner (Philips, Netherlands) using an anterior torso coil array in combination with a posterior spine coil array with 28 receiver channels. Both, adult and fetal PC-MRI sequences were acquired with an in-plane resolution of 1.25 mm × 1.25 mm and a slice thickness of 5 mm; FOV 240 mm × 240 mm; flip angle 20°. TR and TE were set to shortest, resulting in TR = 8.4 ms and TE = 5.2 ms in the fetal cases. Adult PC-MRI sequences were acquired on the common carotid artery and at the jugular veins in the same slice, as in previous validation experiments by Seed et al.^15^. Measurements were taken bilaterally. For fetal data, PC-MRI images were acquired in six fetal vessels: the ascending aorta (AAO), descending aorta (DAO), superior vena cava (SVC), main pulmonary artery (MPA), ductus arteriosus (DA) and the umbilical vein (UV) in slice positions suggested by Jansz et al.^7^.

To adapt for the lower HRs in adults the TR was increased to maintain the temporal resolution as for a fetal scan. One average and 4 views per segment were acquired, resulting in a temporal resolution of approximately 65 ms. In non-ECG gated sequences, a virtual electrocardiogram (VCG) was used with a simulated RR-Interval of 545 ms giving around 8.5 cardiac phases which were interpolated into 15 phases in total. In the adult scans the HR was measured by a surface ECG first, to then set the VCG HR below to ensure an oversampling of the cardiac cycle. Total scan time was roughly 40–60 s, depending on the HR of the subject. Velocity encoding (Venc) settings depended on the attempted vessel and varied between 50–150 cm/s. All fetal acquisitions were performed with a specific absorption rate of < 0.1 W/kg and limited noise and PNS levels.

**Figure 1:**
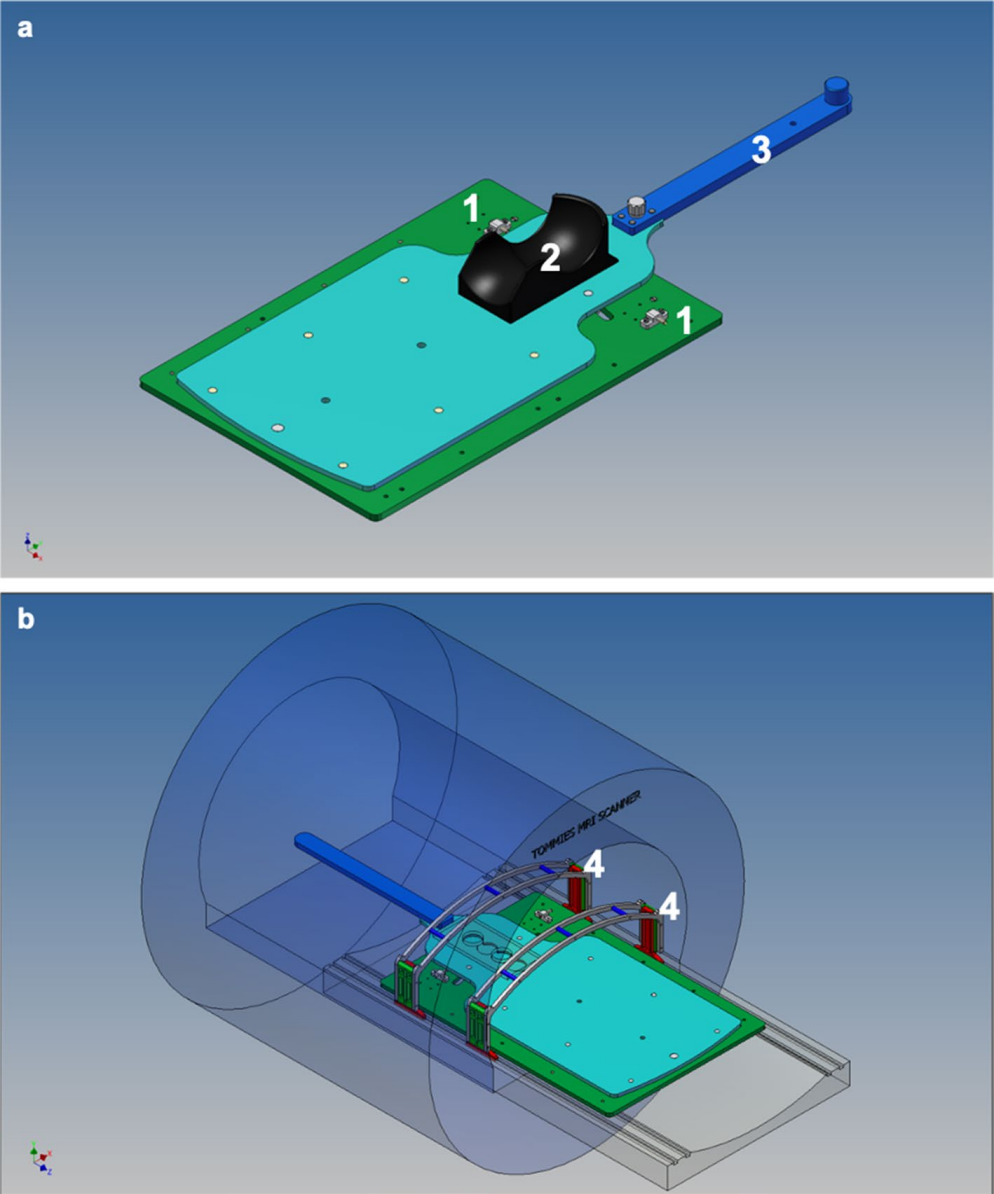
**a** (top) and **b** (bottom) Construction design of the motion device. (**a**) Detailed overview of the main components of the device: flat upper plate (light blue), which is mobile and moves the volunteer who is placed on it. Lower sub-plate (green), which is stationary fixed on the patient table. Integrated mechanical stops limiting the translational range of motion (1). Headrest to immobilize the neck of the volunteer (2). Elongated handle to perform the motion of the upper plate (3) while a rail is guiding the movement of the upper plate on the lower plate. Central pin connecting the upper and lower plate and representing the centre of rotation. (**b**) Fitting of the motion device into an Ingenia MRI-scanner. Coil bridges simulating the belly top of a pregnant woman and ensuring that the anterior body coil stays stationary and contact free above the moving volunteer below (4).

To simulate fetal motion in a reproducible way in adult volunteers a bespoke motion simulation device was used. The device introduces controlled side to side movement of the neck vessels with minimum physiological stress. A detailed overview on the device and its mode of operation is displayed in Fig. 1. During experiments the motion is performed by a person positioned at the end of the magnet using the elongated handle of the device.

The mechanical movement of the motion device was validated in advance by measuring the shifts set to 10 mm within 60 s in ten consecutive attempts.

The speed, translational offset and frequency of simulated fetal motion was estimated from previously published data^20–22^. According to previous studies, the speed of fetal motion is generally between 0.25–2 cm/s with a translational offset ranging from 2.8–15.5 mm, and a frequency of less than 9 movements per 60 s. In order to test the effect of motion during different times of the scan we elected to divide the scan into three equal time periods. As we used Cartesian acquisition, the first and last third therefore corresponded to the outer sections of the k-space, whereas the middle third corresponded mostly to the acquisition of the centre of k-space. In each of these three sections, simulated fetal motion was performed continuously throughout the whole section. Due to the relatively low adult HR compared to a fetus, the speed of motion was scaled by an estimated HR of an adult to ensure the same displacement (in mm) per RR-interval as in a fetus. The relationship is described as in the equation below.

$$\frac{110\, bpm}{{HR\, input\, for\, adult\, scan}} = \frac{motion\, speed\, in\, fetus}{{motion\, speed\, in\, adult\, scan}}.$$

This resulted in the definition of two speeds: slow motion at 0.25 cm/s and fast motion at 0.5 cm/s.

While the mechanical stimulus by the motion device was the same for each volunteer, we applied a retrospective rescaling to the speed and the displacement of motion to take individual anatomy and HRs into account. This is later referred to as corrected speed and displacement and is in concordance with physiological fetal values. The rescaling of motion speed was performed by using the equation above and the corrected displacements.

The translational displacement was rescaled by measuring the shift of the carotid artery in between two individual BTFE-images in the outer positions of the motion device at the beginning and end of the protocol.

Each measurement was repeated twice, with a further series without motion acquired at the beginning (baseline) and at the end (control) of the whole protocol.

The gating for both, fetal and adult data was performed retrospectively with the MOG-Public Software 2.6 (https://github.com/MetricOptimizedGating/MOG-Public) to detect the subject’s HR. The gated images were then converted into DICOM format with an inhouse built tool for MATLAB (MathWorks, US, Version R2017b). The fetal and the adult flow data was measured in a commercially available clinical cardiac MRI software package (CVI42, Circle Cardiovascular Imaging, Calgary, Version 5.6.4) using semi-automated contouring techniques integrated into the software following an internally validated protocol. No background correction was applied. Flow sequences of five randomly selected adult volunteers were measured by a blinded and independent second observer and the interobserver variability was calculated. All unprocessed fetal flow sequences were again scored and afterwards measured by two blinded observers and interobserver variability was calculated.

**Figure 2:**
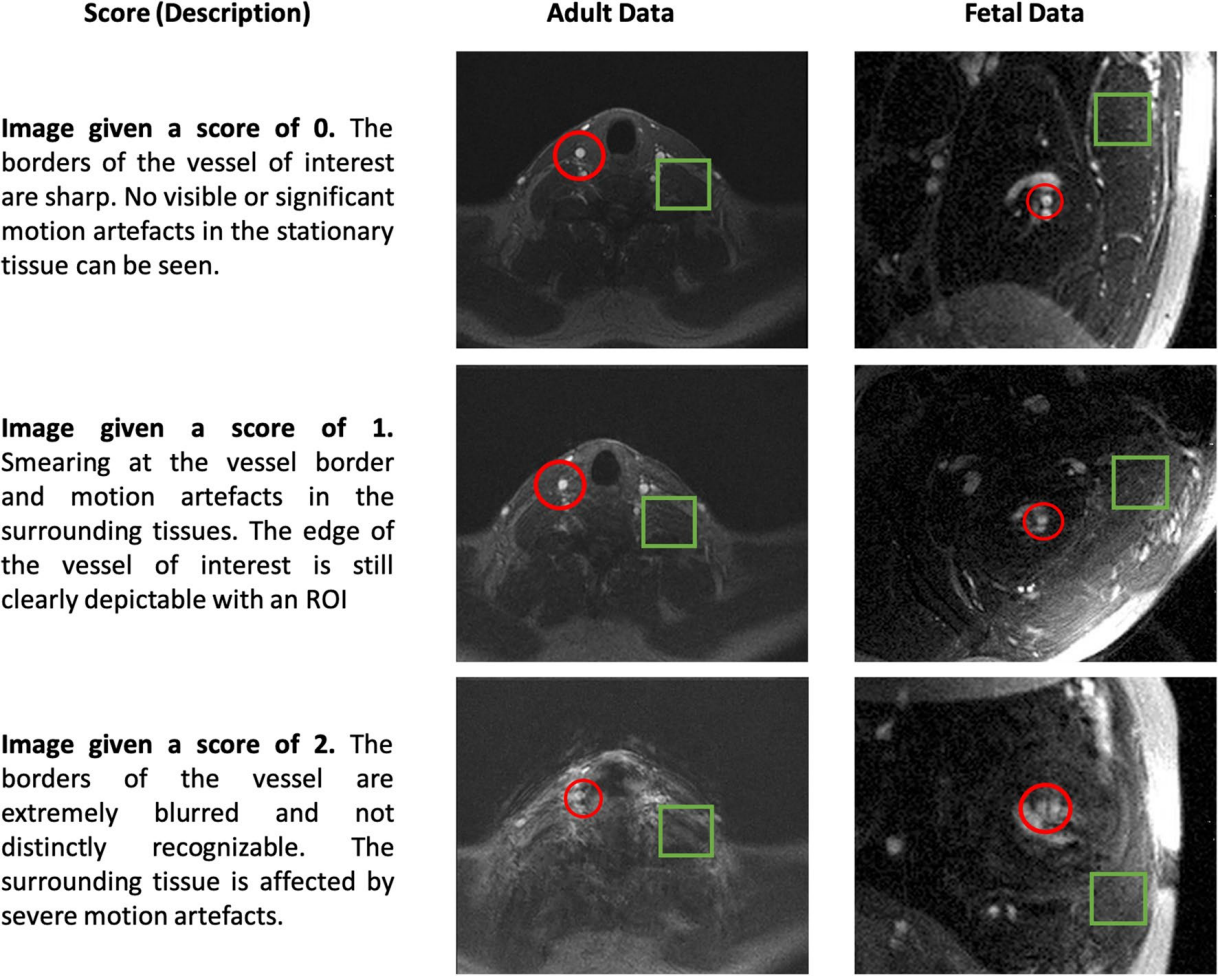
Examples for images prior to the MOG reconstruction. Displayed are examples of images given different scores in fetal scans and in adult volunteers. The red circle indicates the area of interest with the vessel of interest. For the fetus, scans of the AAO are presented, in the adult scans the CA is shown. The green square marks an area of stationary tissue potentially corrupted with motion artefacts.

A three-point scoring system was established for rating motion artefacts in non-gated raw images prior to MOG reconstruction. An overview on the criteria and examples of representative images of each score are displayed in Fig. 2. All acquired images were randomised and scoring was performed on 2D image magnitude sequences.

### Statistical analysis

If not stated otherwise, the results are presented as mean ± standard deviation (SD) or median ± inter quartile range depending on the distribution. Statistical analysis was performed using SPSS (IBM Corp, USA, Version 25). Graphs were plotted with GraphPad Prism (GraphPad Software, Version 8). The error produced by the impact of motion is shown as relative offset of the measurement to the reference scan without motion in mean (median) difference in percent ± SD (inter quartile range) and was calculated by using a one-way ANOVA with a Dunnett’s multiple comparison test. Differences within the repeats of an acquisition were tested with a paired t-test. Inter-observer variation was calculated with the ICC. CoV was calculated as SD divided by mean of differences, displayed in percent.

### Ethics approval and consent to participate

All scans were made in concordance to an ethical approval in advance. The consenting and scans of adult volunteers were done under the REC: 01/11/12/ approved by the London Bridge Research Ethics Committee. The scans and recruitment of pregnant mothers were in accordance to the ethics of long-running fetal imaging studies with REC 14/LO/1806 approved by the same Ethics Committee as above and with REC 07/H0707/105 approved by West London & GTAC Research Ethics Committee.

## Results

### Simulated motion experiment

For the motion study 10 healthy volunteers were prospectively recruited and consented. The mean age was 27.7 years (19–34 years, six female, four male). The mean VCG HR was 55.5 bpm (range 40–75). The carotid artery and the jugular vein were imaged successfully on both sides of the neck in all 160 studies. In one volunteer, fast motion simulation could not be obtained due to limited scan time; however, all other datasets were completed successfully. Two other scans corrupted by motion in the middle section could not be gated due to severe motion artefacts and had to be excluded. MOG was successfully performed in the remaining 155 scans. The jugular vein was massively corrupted in two scans during motion in the middle section that no flows could be measured. In one volunteer no reasonable flows of the jugular veins could be acquired due to an incidental finding of an absent jugular vein with multiple small venous collaterals.

Reproducible fetal motion could be simulated in the desired ranges successfully. The accuracy of the mechanical speed of the motion device within 10 repeats was 0.251 cm/s (± 0.01 cm/s) for slow motion and 0.501 cm/s (± 0.01 cm/s) for fast motion respectively. Full details on the mean corrected amplitude and velocity of the movement, as well as the CoV for both repeats of each acquisition are presented in Table 1. The HR detected by MOG was significantly lower for scans corrupted with slow motion during the first and last section of the scan compared to the surface ECG (mean difference 1.95/min ± 2.2/min; p = 0.02 in the first section, 1.4/min ± 1.6/min; p = 0.02 in the last section respectively). Simulated motion during the middle section caused a significant misdetection of the HR by MOG during fast motion (mean difference 4.9/min ± 4.9/min; p = 0.01), whereas corruption with slow motion did not have a significant impact on MOG (mean difference 1.6/min ± 8.8/min; p = 0.58). The HR detected by MOG did not show significant differences in between each section. Simulated motion during the middle section of an acquisition resulted in lower volume flow rates over time compared to motion free acquisitions and to acquisitions with motion corruption during the outer two sections as shown in Table 1.

**Table 1:** Overview on measurements corrupted with motion on different timepoints of the scan (sections).

Timepoint and speed of motion	No motion		First section		Middle section		Last section	
	Baseline	Control	Slow	Fast	Slow	Fast	Slow	Fast
Mean heart rate (± SD) by ECG in 1/min	64.2 (± 10.6)	63.0 (± 9.4)	63.3 (± 10.1)	63,4 (± 9,5)	62.4 (± 9.4)	62.8 (± 9.2)	63.1(10.5)	62.7 (± 9.5)
Number of scans with successful MOG/total number of scans available for MOG	20/20	20/20	20/20	19/19	18/20	19/19	20/20	19/19
Mean heart rate (± SD) by MOG in 1/min	64.5 (± 10.9)	63.1 (± 9.1)	61.35 (± 10.7)	63.1 (± 8.6)	60.8 (± 12.9)	57.85 (± 7.0)	61.7 (± 11.3)	62.25 (± 9.1)
Difference between heart rate by ECG and MOG (± SD) in 1/min; p-value	− 0.3 (± 4.3); 0.83	− 0.1 (± 1.4); 0.91	**1.95 (± 2.2); 0.02**	0.3 (± 2.1); 0.66	1.6 (± 8.8); 0.58	**4.9 (± 4.9); 0.01**	**1.4 (± 1.6); 0.02**	0.45 (± 1.2); 0.28
Corrected motion amplitude (range) in mm/variability (± SD) over the protocol in mm	11.2 (9.4–13.8)/0.04 (± 0.39)							
Corrected motion speed (cm/s)	*–*		0.57 (± 0.13)	1.15 (± 0.27)	0.57 (± 0.13)	1.15 (± 0.27)	0.57 (± 0.13)	1.15 (± 0.27)
Successful measurements of the carotid artery/total scans available after MOG	20/20	20/20	20/20	19/19	18/18	19/19	20/20	19/19
Carotid artery: mean flow rate (ml/kg/min)	4.2 (± 1.1)	4.2 (± 1.2)	4.3 (± 1.1)	4.2 (1.2)	2.5 (± 1.1)	2.8 (± 1.1)	4.2 (± 1.1)	4.2 (± 1.1)
Carotid artery: mean difference from baseline (± SD) in %	–	− 0.3 (± 7.3)	1.7 (± 6.4)	0.8 (± 6.9)	− **39.1 (± 20.6)**	− **32.1 (± 17.9)**	0.8 (± 4.2)	1.0 (± 6.5)
Carotid artery: p-values	–	0.9997	0.7109	0.9945	**< 0.0001**	**< 0.0001**	0.9215	0.969
Carotid artery: mean flow difference between fast and slow motion (± SD) in %; p-value	–		1.0 (± 3.9); 0.27		**6.9 (± 14.3); 0.04**		0.2 (± 3.8); 0.81	
Successful measurements of the jugular vein/total scans available after MOG	19/20	19/20	19/20	18/19	16/18	17/19	19/20	18/19
Jugular vein: mean flow rate (ml/kg/min)	3.1 (± 1.1)	3.1 (± 2.1)	3.1 (± 2.0)	3.0 (± 2.2)	2.2 (± 1.6)	1.8 (± 1.8)	3.1 (± 2.2)	3.1 (± 2.2)
Jugular vein: mean difference from baseline (± SD) in %	–	− 5.8 (± 14.1)	− 1.6 (± 21.0)	− 4.2 (± 19.5)	− **36.1 (± 33.1)**	− **47.0 (± 26.9)**	− 0.9 (± 12.8)	− 5.6 (± 25.1)
Jugular vein: p-values	–	0.4265	0.9996	0.9048	**0.0015**	**< 0.0001**	0.9996	0.8797
Jugular vein: mean flow difference between fast and slow motion (± SD) in %; p-value	–		− 5.0 (± 27.7); 0.47		− 10.9 (± 37); 0.23		− 4.7 (± 20.8); 0.35	
CoV (± SD) of two repeats in %	5.9 (± 11.3)	6.4 (± 8.7)	5.6 (± 8.4)	7.1 (± 14.2)	32.5 (± 35.5)	25.0 (± 31.0)	5.7 (± 7.2)	4.9 (± 9.6)
Mean score of raw image data	0	0	0.9	0.8	2	2	0.8	0.9

*CoV* coefficient of variation, *SD* standard deviation.

Significant values are in bold.

**Figure 3:**
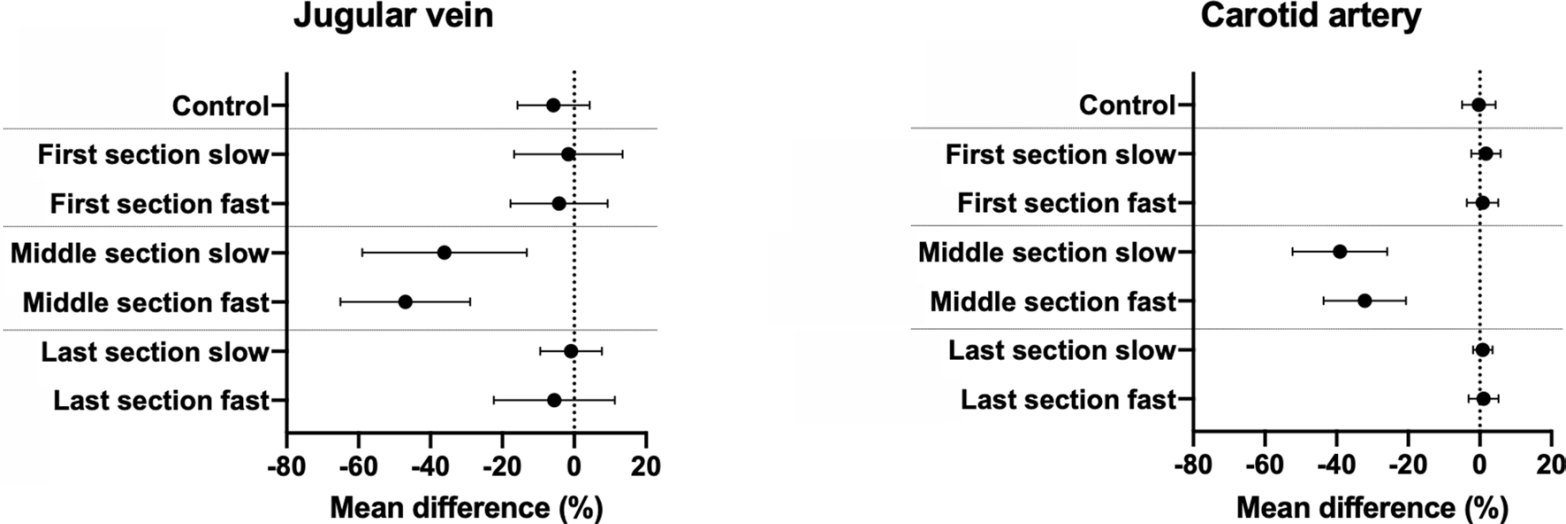
Impact of motion corruption in each section of the scan. The graphs show the mean differences (± SD) between the baseline (motion free) acquisition and each category of motion corrupted acquisition for the jugular vein (left) and carotid artery (right). Acquisitions corrupted with motion during the middle section showed significantly lower blood flow measurements compared to the baseline scan. However, acquisitions corrupted with motion in the other two sections, showed no significant bias compared to the baseline scan.

In the carotid artery the mean relative difference between the baseline and the control (both motion free) scans was − 0.3% (95% Confidence interval (CI) − 5.0 to 4.4%; p = 0.99). Scans corrupted with motion in the first and in the last section showed no significant difference in blood flow measurements compared to measurements in the baseline scan without motion [1.1% (95% CI − 0.5% to − 2.7%; p = 0.27)]. For scans corrupted with motion in the middle section blood flow measurements were significantly lower compared to measurements in the baseline scan [− 35.6% (95% CI − 42.2% to − 29.1%; p < 0.0001)]. For the jugular vein the mean difference in blood flow measurements between the baseline and the control scans was − 5.8% (95% CI − 15.8% to − 4.3%; p = 0.43). Blood flow measurements in acquisitions corrupted with motion in the first and the last section of the scan showed no significant difference compared to the quantifications in the baseline scan [− 3.1% (95% CI − 8.7% to 2.6%; p = 0.44)]. Blood flow volumes in the jugular vein from scans with motion corruption in the middle section were significantly lower compared to those obtained at baseline scans [− 41.6% (95% CI − 52.4% to − 30.7%; p < 0.0001)]. A summary of these findings is shown in Fig. 3. The speed of the motion (fast/slow) had a significant impact on flow measurements in the carotid artery during motion in the middle section only (p < 0.05); however, we could not show any impact of the speed of motion on flow measurements of any other vessels in this study.

**Figure 4:**
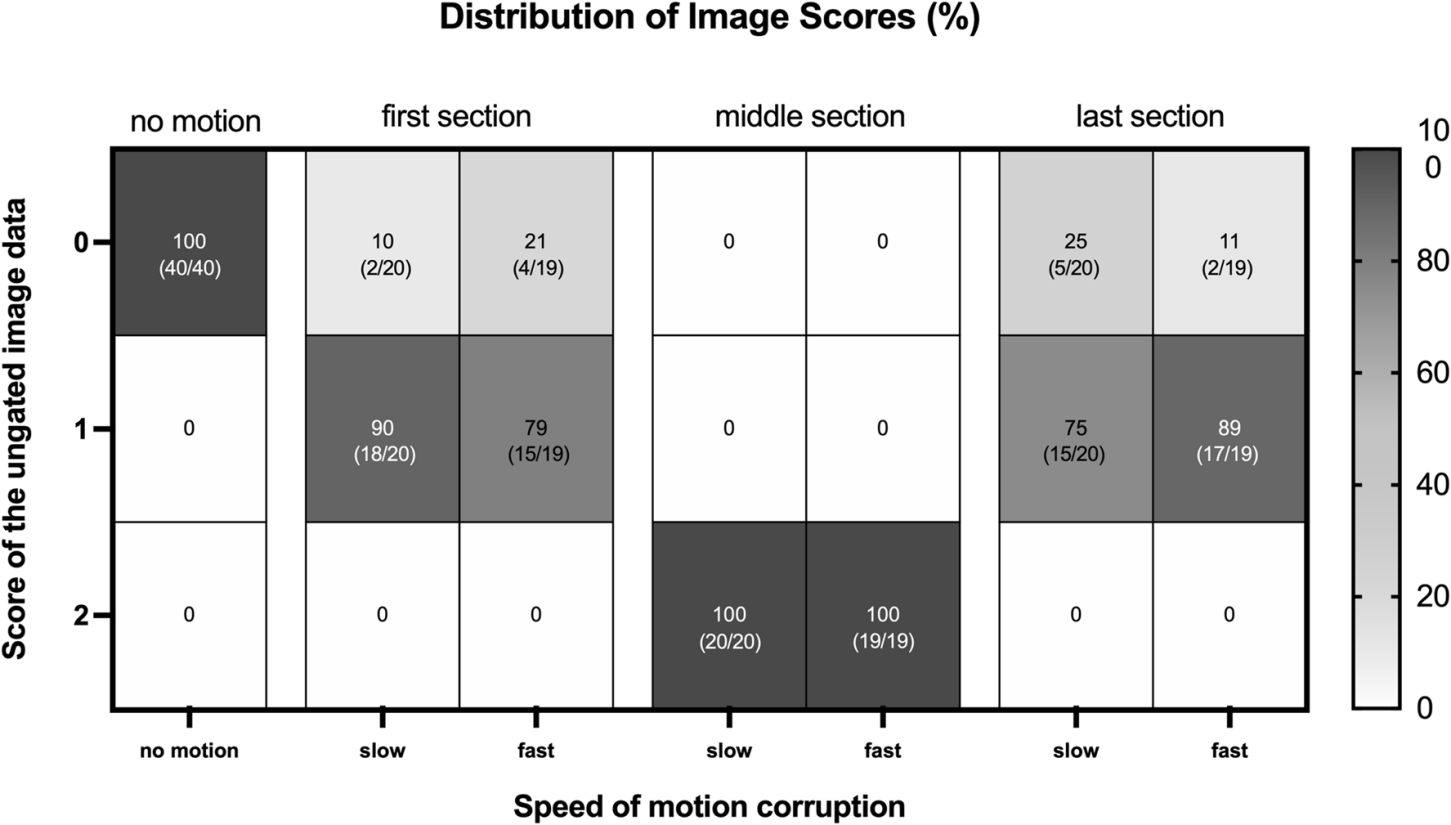
Image scores of scans with motion corruption in each section. Displayed is the absolute and relative number (%) of ungated images rated with a score of zero, one or two sorted by the timepoint (section) and the speed of the corrupting motion. On the righthand side a scale bar shows the matching colour for the referring values in percent. All images without motion were labelled with a score of zero (100%) whereas all images containing motion corruption in the middle section received a score of two (100%). Images with motion in the first or last section were mostly rated with a score of one. None of the images in the first or last section of the scan was rated with a score of two.

**Figure 5:**
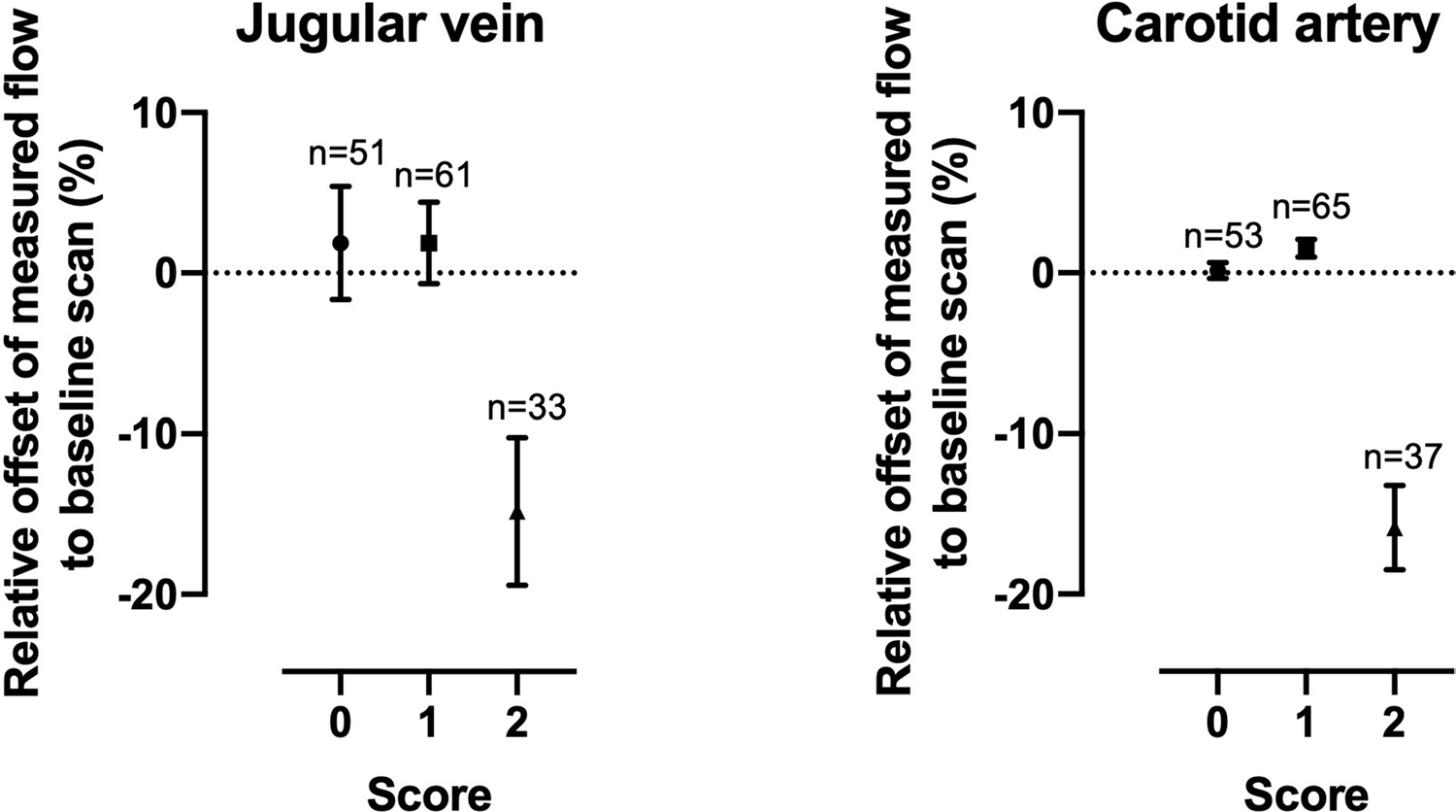
Impact of motion on flow-measurements in images with different scores. The relative deviation in percent (offset) of a flow measurement compared to its reference (baseline) scan without motion as a function of the given score for its referring image. The dots display the mean ± 95% Confidence Interval. The number of scans rated with an individual score is displayed above the referring score. In both types of vessels, scans scored with a score of two had significantly lower flow compared to scans with a score of zero (p < 0.0001). Scans with a score of one showed no significant bias in their flow measurements compared to scans with a score of zero.

For the MRI-scans of the adult volunteers, image scoring was successfully performed in all 155 available images. The distribution of scores is shown Fig. 4 with the mean difference of each individual flow measurement compared to the reference scan in Fig. 5. We could not show any significant difference between the measured flows of images rated with a score of 0 or 1 (referring baseline measurement vs measurements with scores of 0 or 1; p > 0.16). However, flow measurements in images with a score of 2 showed significantly lower blood flow then their baseline scan (referring baseline measurement vs measurements with scores of 2; p < 0.0001).

Regarding the interobserver variability in adult data, images rated with a score of 0 and 1 had a very good ICC for flow measurements in the carotid artery and the jugular vein. However, flow measurements of the carotid artery and the jugular vein in images with a score of 2 had a significantly higher inter-observer bias (p < 0.0001) with a lower ICC in the carotid artery and in the jugular vein compared to measurements in images with a score of zero and one (compare Table 2).

**Table 2:** Inter-observer variability in venous and arterial flow measurements in adult and fetal vessels.

Adult measurements	Carotid artery			Jugular vein		
Image score	0	1	2	0	1	2
Number of flow measurements	60	60	36	56	50	33
Mean flow difference in flow measurements of the observers ± SD (%)	7.1 ± 4.2	8.5 ± 5.7	**39.7 ± 34.2**	3.2 ± 2.6	5.8 ± 6.0	**51.6 ± 55.6**
ICC (95% CI)	0.99 (0.98–0.99)	0.98 (0.96–0.99)	**0.81 (0.64–0.90)**	0.99 (0.99–1.0)	0.99 (0.99–1.0)	**0.90 (0.81–0.95)**
Significance of the difference—compared to scans rated with zero	–	p = 0.86	**p < 0.0001**	–	p = 0.81	**p < 0.0001**

Fetal measurements	Artery			Vein		
Image score	0	1	2	0	1	2
Number of flow measurements	3	23	12	5	7	8
Mean flow difference in flow measurements of the observers ± SD (%)	4.3 ± 4.8	5.8 ± 5.1	**26.0 ± 23.4**	4.2 ± 4.4	5.3 ± 3.1	**28.5 ± 22.4**
ICC (95% CI)	0.99 (0.78–1.0)	0.99 (0.97–0.99)	**0.86 (0.51–0.96)**	0.96 (0.65–0.99)	0.99 (0.94–0.99)	**0.76 (− 0.20 to 0.95)**
Significance of the difference—compared to scans rated with zero	–	p = 0.96	**p = 0.03**	–	p = 0.99	**p = 0.02**

*ICC* intra class correlation, *SD* standard deviation, *CI* confidence interval.

Significant values are in bold.

### Fetal PC-MRI scoring and interobserver variability

Fetal data was acquired on 10 pregnant women with a mean gestational age of 32 weeks (28–36). Eight pregnant women had a fetus with suspected CHD (seven suspected aortic coarctation, one suspected hypoplastic left heart syndrome) and two were healthy volunteers. Flow measurements were successfully performed in all 10 cases by two observers. No acquisition of the AAO-flow was feasible in the case with suspected hypoplastic left heart syndrome. In one patient no reliable acquisition for the DA could be found to measure blood flow due to severe maternal motion.

**Figure 6:**
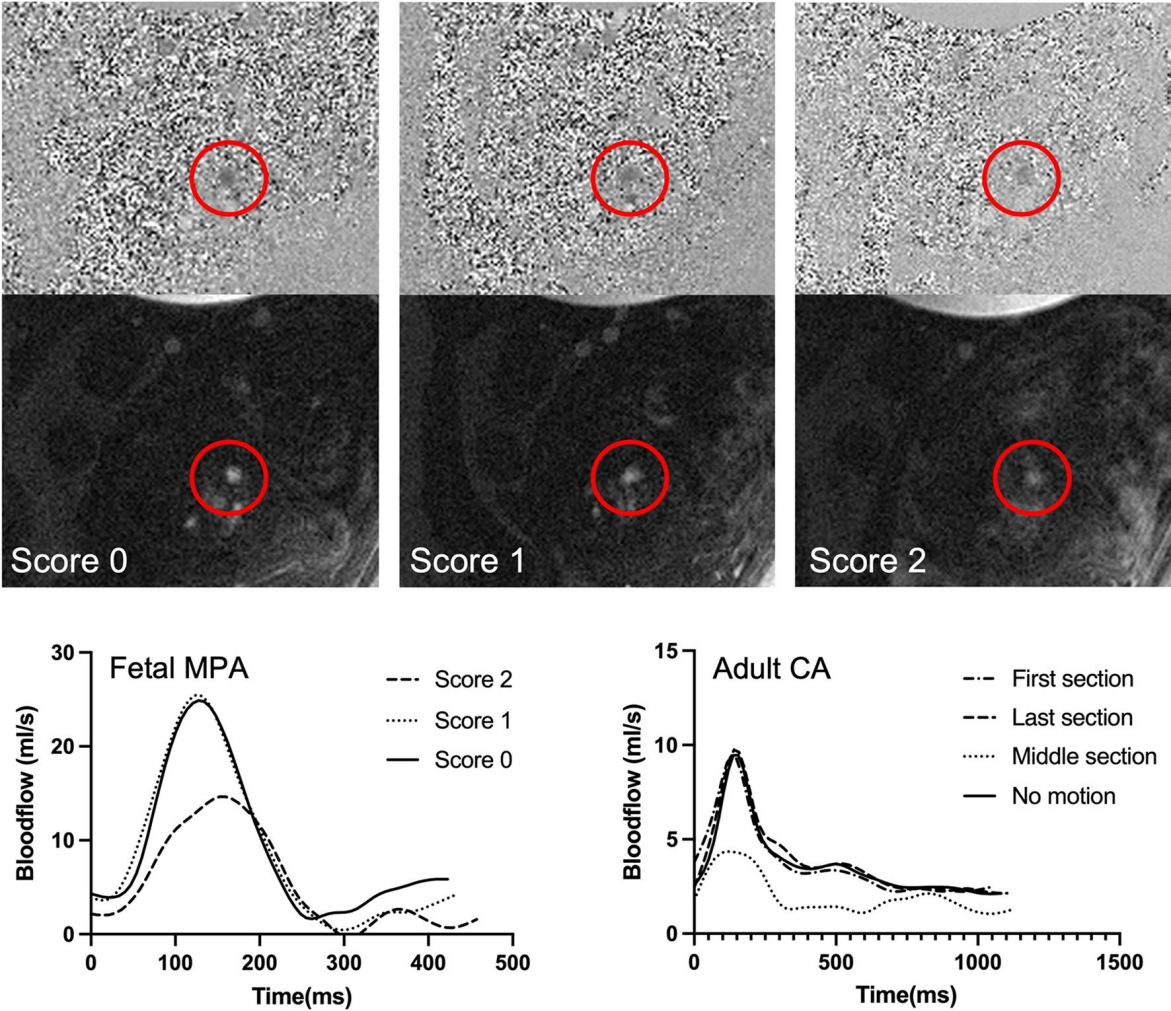
Examples for fetal flow measurements with different scores. Acquisitions of the fetal MPA in the same fetus rated with three different images scores due to different levels of motion corruption. The top row shows the velocity images with the referring magnitude image below. The red circles mark the MPA. The graph at the bottom left shows the flow curves for the acquisitions of the fetal MPA above. On the bottom right, concordant flow curves of measurements in the carotid artery (CA) of an adult during motion corruption in the different sections are displayed.

Representative magnitude and velocity images of three acquisitions of the MPA obtained from the same fetus and rated with different image scores are shown in Fig. 6. Additionally, flow-curves of the referring fetal measurements are displayed next to flow-curves derived from adult scans with motion corruption during different sections of the scan.

Fetal flow measurements in images with a score of 0 and 1 had a significantly lower inter-observer bias (p < 0.05) compared to measurements in images with a score of 2. The corresponding ICC showed very good results in arterial and in venous flow measurements in images with a score of zero, and in images with a score of one. The ICC was lower for arterial flow measurements and for venous flow measurements in fetal images rated with a score of two (compare Table 2).

## Discussion

In this study we have provided a detailed insight into the robustness of MOG-PC blood flow imaging in the face of simulated motion and demonstrated that a simple three-point scoring system accurately identifies important motion-corruption on pre-processed images. In particular, any degree of motion during the middle section of the scan (corresponding to the centre of k-space in Cartesian acquisition)^23^ was found to corrupt flow measurements unpredictably, leading to underestimation of blood flow measurements in adult datasets and poor interobserver reproducibility in both adults and fetal cases. Interestingly, even if significant differences could be observed in HRs detected by MOG compared to the HRs measured by a surface ECG during motion corruption, those differences were rather small in absolute values. The highest misgating could be observed under the face of simulated fast motion in the middle section. However, this difference is relatively small compared to the error in the blood flow measurements. Even if MOG potentially contributes to false flow measurements during fetal motion, the observations are keeping with established knowledge on the nature of Cartesian k-space acquisition^23^, and have been well-described in postnatal PC-MRI acquisitions with conventional ECG-gating^19,24^. Importantly, data corrupted by motion in this way was readily identified using the three-point scoring system described (scoring 2 out of 2 in all cases). Less significant movement during acquisition, scored as 0 or 1 in this system, did not appear to have any appreciable impact on the accuracy of subsequent flow measurements.

The dominant source of the error appears to be an underestimation of peak flow volumes and lower overall flow rates under the presence of motion. Interestingly, the speed of motion within the tested ranges did not have a major impact on the relative flow error in the in-vivo model. In addition, while the impact of motion was less notable in venous compared to arterial vessels, both showed the same trend in their relative error during motion corruption.

The ability to identify motion-corrupted sequences at the time of acquisition has several potential advantages. Earlier studies on fetal cardiovascular flow imaging mentioned that fetal motion frequently causes the need for image reacquisition^2,7,11,25^. A simple three point scoring system, such as the one described in this study, could therefore be a significant aid to real-time decision-making at the time of acquisition, potentially reducing the need for multiple repeat sequences, shortening overall scan time as well and the amount of time required for post-processing^11^. In addition, the majority of studies describing the use of PC-MRI with MOG have been focussed on pregnancy well into the third trimester, in part due an increased likelihood of significant fetal motion at earlier gestations. The ability to robustly assess the degree of fetal motion at the time of acquisition may be a useful tool in applying these methods earlier in pregnancy.

Motion corruption is already shown to be reduced in radial or spiral data acquisitions where the centre of k-space is acquired during all imaging phases^18,26^. First implementations for the application of multidimensional imaging using radial acquisition in fetuses have already been made and showed promising results and improvements of the flow accuracy when adding motion correction techniques in simulated data^25,27,28^. Moreover, three-dimensional motion-corrected techniques have been described to assess static anatomical^9,21^, and more recently functional features of the fetal cardiovascular system^29^. The findings in this study, along with these other advanced imaging techniques, could potentially be important components in compensating for uncontrolled fetal motion in cardiovascular MRI in the future.

### Limitations

The motion device presents a first approach to simulate fetal motion in an in-vivo model.

In reality, fetal motion is more complex and mostly three dimensional including not just in-plane motion from left to right, but also rotational shifts and through plane motion, however, it is likely that significant through plane motion would be recognised at the time of acquisition^21^. This might reduce the applicability of the scoring system as developed in adult scans for fetal acquisitions.

The effect of motion on vessel edge detection for defining the region of interest have not been analysed separately in this experiment, as we expected the information to be included within the blood-flow rates. However, residual artefacts might impact the segmentation during blood flow measurements.

Image quality in fetal scans may be impaired by other factors, such as maternal breathing or other movement, as well other factors affecting MRI signal such as fetal position and maternal habitus. Additionally, smaller vessels will have a broader representation in k-space and therefore might be more sensitive to motion during the outer sections of the acquisition^23^. These factors were not specifically investigated in this study.

Similarly, the speed and consistency fetal motion is likely to vary more than simple two-speed model described in this study. Further studies examining the effects of different types and speeds of fetal movements may offer further insights into the nature of these effects in the future. Due to additional motion this would have invariably produced, we were not able to dial up the adult HR to rates of a fetus with a bicycle as done by Seed et al.^15^ and instead applied a scaling system to correct for this. Finally, all findings apply for Cartesian acquisitions of k-space in PC-imaging only; further studies are needed to determine the utility of this scoring system using other acquisition methods.

## Conclusion

Fetal blood flow measurements in PC-MRI using MOG are robust to fetal motion provided this does not occur during the middle section of Cartesian acquisition. Motion during this period significantly impacts both the accuracy and reproducibility of subsequent flow measurements and can be readily identified on non-processed image data using a simple three-point scoring system.

## Data Availability

The datasets used and/or analysed during the current study are available from the corresponding author on reasonable request.
